# Investigation of extended-gate field-effect transistor pH sensors based on different-temperature-annealed bi-layer MWCNTs-In_2_O_3_ films

**DOI:** 10.1186/1556-276X-9-502

**Published:** 2014-09-16

**Authors:** Shang-Chao Hung, Nai-Jen Cheng, Cheng-Fu Yang, Yuan-Pin Lo

**Affiliations:** 1 Department of Information Technology & Communication, Shih Chien University Kaohsiung Campus, Neimen, Kaohsiung 84550, Taiwan, R.O.C; 2 Institute of Photonics and Communications, National Kaohsiung University of Applied Sciences, Kaohsiung 80778, Taiwan, R.O.C; 3 Department of Chemical and Materials Engineering, National University of Kaohsiung, Kaohsiung 81147, Taiwan, R.O.C; 4 Graduate Institute of Electro-Optical Engineering and Department of Electronic Engineering, National Taiwan University of Science and Technology, Taipei 10608, Taiwan, R.O.C

**Keywords:** Multiwalled carbon nanotubes, pH sensor, Bi-layer, MWCNTs-In_2_O_3_ films

## Abstract

In this paper, indium (In) films were deposited on glass substrates using DC sputtering method. Multiwalled carbon nanotubes (MWCNTs) and dispersant were dissolved in alcohol, and the mixed solution was deposited on the In films using the spray method. The bi-layer MWCNTs-In_2_O_3_ films were annealed at different temperatures (from room temperature to 500°C) in O_2_ atmosphere. The influences of annealing temperature on the characteristics of the bi-layer MWCNTs-In_2_O_3_ films were investigated by scanning electron microscopy, X-ray diffraction pattern, Fourier transform infrared (FT-IR) spectroscopy, and Raman spectroscopy. A separative extended-gate field-effect transistor (EGFET) device combined with a bi-layer MWCNTs-In_2_O_3_ film was constructed as a pH sensor. The influences of different annealing temperatures on the performances of the EGFET-based pH sensors were investigated. We would show that the pH sensitivity was dependent on the thermal oxygenation temperature of the bi-layer MWCNTs-In_2_O_3_ films.

## Background

Carbon nanotubes (CNTs), an important group of nanoscale materials, have received great attention in different fields since their discovery in 1991 by Iijima [1]. Due to their unique structural, electronic, and mechanical properties, CNTs make themselves very attractive materials for a wide range of applications [1-3]. CNTs with their well-defined nanoscale dimensions and unique molecular structure can be used as bridges linking biomolecules to macro/micro-solid-state devices so that bioevent information can be transduced into measurable signals. Among them, chemical and biological sensors [4] based on CNTs have been the target of numerous investigations because of their simplest chemical composition and atomic bonding configuration even though considerable challenges remain in a specific end use. For that, multiple types of CNT-based chemical sensors have been developed for sensing application. Because single-walled carbon nanotube (SWCNT)-field-effect transistors (FETs) offer several advantages for sensing including the ability to amplify the detection signal with the additional gate electrode, Chen et al. used SWCNT-thin-film transistors (TFTs) as gas sensors to detect methyl methylphosphonate, a stimulant of benchmark threats [5]. Also, Karimi et al. proposed an analytical model of graphene-based solution-gated (SG) FETs to constitute an important step towards development of DNA biosensors with high sensitivity and selectivity [6]. Dong et al. fabricated carbon monoxide (CO) and ammonia (NH_3_) gas sensors using interdigitated electrodes on Si wafer, and they found that 10 ppm of CO and NH_3_ could be electrically detected using a carboxylic acid-functionalized single-walled carbon nanotube (C-SWCNT) [7].

Those researches prove that semiconductor active devices have been developed for sensing application, and SWCNT-FETs offer several advantages for sensing including the ability to amplify detection signals [8]. In the past, CNTs can also be used to investigate as a pH sensor. For example, Kwon et al. fabricated a simple and fast-response pH sensor composed of SWCNTs using a non-vacuum spray method [9]. An ion-sensitive field-effect transistor (ISFET) device is applied to an electrochemical sensing device, and the structure of a separative extended-gate field-effect transistor (EGFET) device has been developed from the ISFET device. Thus, an EGFET device is also a semiconductor active device with a different structure to produce FET isolation from the chemical environment, in which a chemically sensitive membrane is deposited on the end of a signal line extended from the FET gate electrode [10]. The EGFET device's structure also comprises a metal-oxide-semiconductor field-effect transistor (MOSFET) which retains a metal gate electrode and utilizes a signal wire to connect the separative ion sensing films and the field-effect transistor. For that, the EGFET devices can solve the packaging and maintaining problems of ISFET devices, and the EGFET devices can operate at a higher stable condition. The ISFET devices can also be designed from discarded biosensors (the ion sensing films) to save money because they combine two different parts, the sensors and MOSFET. For that, a novel concept combining bi-layer multiwalled carbon nanotubes (MWCNTs)-In_2_O_3_ films and EGFET is proposed for pH sensing application. In this study, the bi-layer MWCNTs-In_2_O_3_ films were used to fabricate the sensing layer and to catch the ions in the solution and EGFET devices were investigated and used to transport the ions while the EGFET device was active. The bi-layer MWCNTs-In_2_O_3_ films were annealed at different temperatures (200°C ~ 500°C), and the effect of annealing temperatures on the characteristics of In_2_O_3_ films and on the performances of pH sensors was investigated.

## Methods

**Figure 1:**
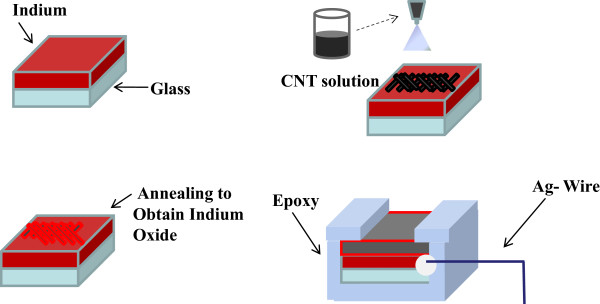
**Fabrication process flow for the formation of MWCNTs/In**_**2**_**O**_**3 **_**sensing layer.**

The detailed process of the fabrication is illustrated in Figure 1. At first, indium (In) films were deposited on glass substrates by RF magnetron sputtering using a pure indium target with purity higher than 99.999% for 1 h. To prepare the In films, the target was pre-sputtered with a DC power of 20 W for 30 min before deposition. The glass substrates were cleaned with standard RCA cleaning processes to remove the native oxide and particles. They were cleaned with acetone, isopropyl alcohol, and distilled water. Deposition of indium films on glass substrates was then performed at room temperature (RT) in a pure Ar (99.999%) ambient with a 2-in. 99.99% purity indium metal target by RF magnetron sputtering for 1 h under the chamber pressure 2.0 × 10^-2^ Torr, flow rate of Ar gas 20 sccm (standard cubic centimeter per minute), and RF power 100 W.

Secondly, 2 mg of as-received MWCNT (Iljin Nanotech Co. Ltd., Seoul, South Korea, average diameter 30 nm) powder with 10 mg of dispersant (type: PVP K30) was ultrasonically dispersed in 10 ml of anhydrous ethanol for 30 min. The solution was spread on In films to form the bi-layer MWCNTs-In films. MWCNT-based suspension was then sprayed on the In-coated glass substrates maintained at 90°C for 40 min by using a portable air spray gun with a distance of 10 cm keeping 3 s with an interval of 1 min for 40 min. The prepared samples were put in the vacuum chamber with 20 mTorr, and N_2_ with 100 sccm was introduced during the temperature raising process. The composite MWCNTs-In films were annealed at different temperatures, ranging from 200°C to 500°C for 1 h. The surface morphology, microstructure, and cross section of the bi-layer MWCNTs-In_2_O_3_ films were characterized by field-emission scanning electron microscopy (FESEM). If the bi-layer MWCNTs-In_2_O_3_ films were annealed at a temperature higher than 500°C, the In_2_O_3_ (In) films were melted. For that, the bi-layer MWCNTs-In_2_O_3_ films could not be annealed at a temperature higher than 500°C. As the temperature was raised to annealing temperature, the chamber was kept at 20 mTorr and O_2_ with 10 sccm was introduced during the annealing process. The addition of O_2_ was used to anneal In into In_2_O_3_. Fourier transform infrared (FT-IR) spectrum was recorded over the range 400 to 1,000 cm^-1^ on a Thermo-Nicolet Avatar 370 FT-IR spectrometer (Thermo Fisher Scientific, Waltham, MA, USA) using the KBr pellet method for the inspected In-O phonon vibration mode measurement. X-ray diffraction (XRD) pattern with Cu Kα radiation (*λ* = 1.5418 Å) was used to find the crystalline structure of In_2_O_3_ films, and Raman measurement obtained from red laser (785 nm) was used to examine the chemical composition of MWCNTs.

**Figure 2:**
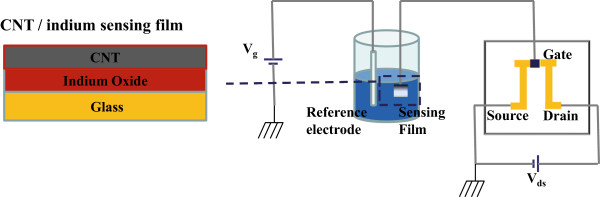
Schematic drawings of the experimental setup and the fabricated EGFET device.

The sensing layer of the designed EGFET devices was fabricated using the bi-layer MWCNTs-In_2_O_3_ films. Figure 2 shows a schematic drawing of the experimental setup of the designed pH sensors. The EGFET devices connected two differently independent structures, one was a sensing structure containing the surface of the sensitive layer and the other was an n-type MOSFET (FET IC4007) structure (Fuji Semiconductors, Tokyo, Japan). The sensing window of pH sensors was 5 mm × 5 mm encapsulated using epoxy with a silver wire connected to the gate of the commercially available n-type MOSFET, which was connected to a Keithley 237 current-voltage meter (Keithley Instruments, Inc., Cleveland, OH, USA). It should be noted that the reference electrode voltage was increased from 0 to 3 V and the drain-source voltage (*V*_DS_) was maintained to be constant at 0.3 V while the drain current was measured.

## Results and discussion

**Figure 3:**
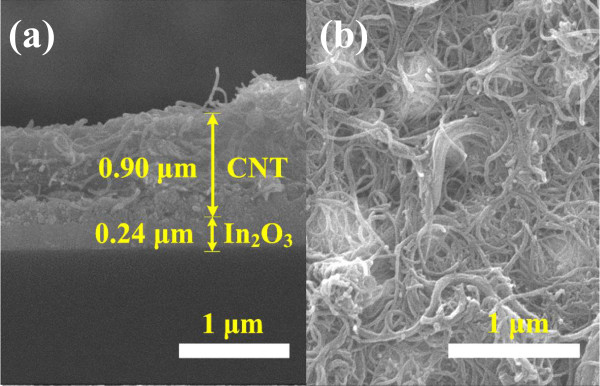
**FESEM images of the MWCNT/In**_**2**_**O**_**3 **_**composite films: (a) cross section and (b) top view.**

Figure 3a,b shows typical top-view and cross-sectional FESEM images of the bi-layer MWCNTs-In_2_O_3_ film; the MWCNTs were formed through spread method, and the bi-layer MWCNTs-In_2_O_3_ film was post-annealed at 400°C. The images show that the MWCNTs adhered firmly on the In_2_O_3_ film, the thickness of the In_2_O_3_ film was about 240 nm (0.24 μm), and the thickness of the MWCNT film was in the range of 0.85 ~ 1.10 μm. MWCNTs are well known for their excellent electrical, mechanical, and thermal properties. Therefore, MWCNTs are good candidates for the manufacturing of small devices or sensors with a special function. In this study, even the thickness of the MWCNTs is not uniform; 0.85 ~ 1.10 μm is enough to sense ions in the liquid solution being tested. The FESEM images also show that the In_2_O_3_ film showed a densified structure and the MWCNTs had large pores. Figure 3 proves that the bi-layer MWCNTs-In_2_O_3_ films can be used as pH sensors.

**Figure 4:**
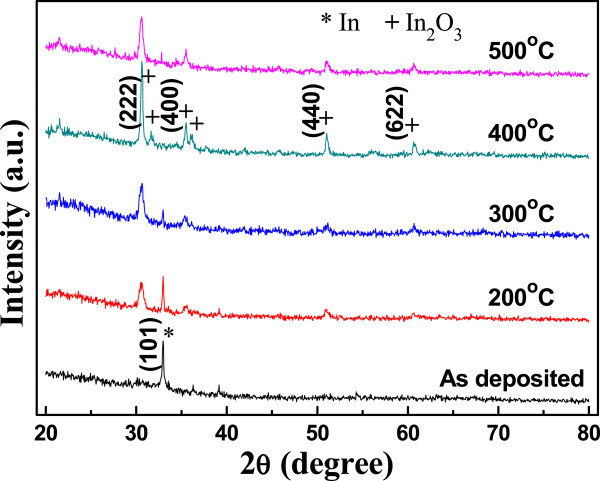
**XRD patterns of MWCNTs/In**_**2**_**O**_**3 **_**films with different annealing temperatures.**

XRD patterns were measured from the as-deposited In films and In films annealed at different temperatures (200°C, 300°C, 400°C, and 500°C), and the results are shown in Figure 4. From the XRD pattern of as-deposited In films, only one strong XRD peak located at around 2*θ* = 32.9° was observed, and this peak was referred to the orientation of the (101) peak (JCPDS card no. 85-1409) for metal In. As the annealing temperature was increased to 200°C, the (222) peak at around 2*θ* = 30.58° for In_2_O_3_ (JCPDS card no. 44-1087) was also observed and the diffraction intensity of the (101) peak for In decreased apparently. As the annealing temperature was 300°C, the diffraction intensity of the main diffraction (101) peak of In phase critically decreased and the diffraction intensity of the main diffraction (222) peak of In_2_O_3_ phase critically increased, and the mainly crystalline peak was (222) of In_2_O_3_ phase rather than (101) of In phase. As the annealing temperature was equal to and higher than 400°C, the (101) peak for In phase was not observed, and (222), (400), (440), and (622) peaks of In_2_O_3_ phase were clearly observed, accompanying two unapparent diffraction peaks at 2*θ* of around 31.2° and 36.5°. The sharpness and diffraction intensity of the (222) peak increased with increasing annealing temperature. The full width at half maximum (FWHM) value of the (222) peak for In_2_O_3_ phase located at around 2*θ* = 30.58° was 0.467°, 0.352°, and 0.454°, respectively, for the 300°C-, 400°C-, and 500°C-annealed In_2_O_3_ films. For 300°C-annealed In_2_O_3_ films, the larger FWHM value is caused by the residual of In. Such a smaller FWHM value implies that as 400°C is used as the annealing temperature, the In_2_O_3_ films have better crystallization results as compared with samples annealed at other temperatures. The phenomena are attributed to the enhanced thermal energy of In_2_O_3_ crystallization as we increased the temperature during oxidation. As we know, a higher annealing temperature can provide more thermal energy to the In_2_O_3_ films for crystallization and the crystal quality is then improved [11]. Figure 4 also shows that 500°C-annealed In_2_O_3_ films had a larger FWHM value and smaller diffraction intensity of the (222) peak; 500°C is too high and the melting of In_2_O_3_ films is believed to be reason for causing this result.

**Figure 5:**
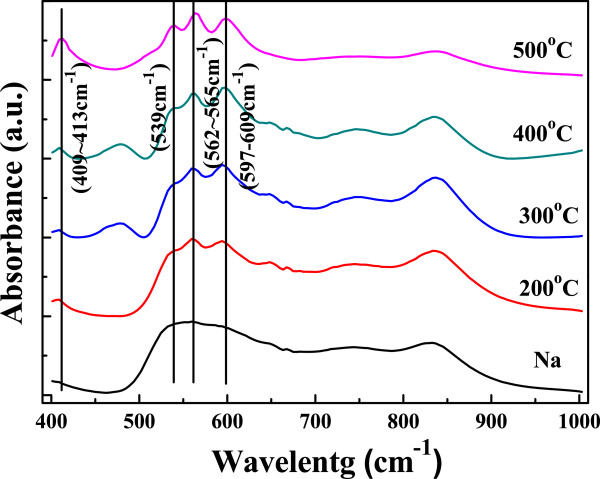
**FT-IR spectra of In**_**2**_**O**_**3 **_**film/glass with various oxygenation temperatures.**

It is well known that vibrational spectroscopy is a very useful technique for the determination of the crystal phase of In_2_O_3_. FT-IR spectra characterization was carried out to get further information on the material composition and structural characteristics of the oxidized In_2_O_3_ films synthesized under various annealing temperatures, and the results are shown in Figure 5. As Figure 5 shows, the FT-IR spectra of samples thermally treated at different temperatures (200°C to 500°C) are quite similar. Four main intense peaks centered at around 600, 565, 539, and 413 cm^-1^ were observed clearly as thermal treatment was over 300°C. According to the previous results reported in the literature, the observed bands at 413 and 557 cm^-1^ are attributed to In-O stretching in cubic In_2_O_3_ whereas the band at 602 cm^-1^ is the characteristic of In-O bending vibrations in In_2_O_3_[12-14]. Also, the appearance of three bands peaking at 540, 565, and 600 cm^-1^ can be assigned to the phonon vibration of In-O bonds [15], which indicates the formation of cubic In_2_O_3_. These FT-IR results have good agreement with the XRD analysis experiment results shown in Figure 4.

**Figure 6:**
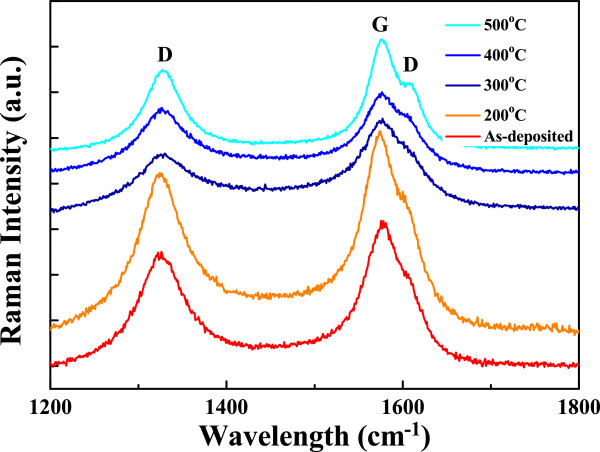
Changes in the relative intensity of D bands compared to that of G bands vs. treatment temperature.

The Raman spectra of as-received nanotubes were recorded using excitation wavelengths in the near-IR range for a study on the effects of wavelength variation. Figure 6 depicts the intensity ratios of the D band over the G band for the MWCNTs in different oxidation temperatures, and Table 1 shows the wavelengths of the *I*_D_ and *I*_G_ peaks and the calculation value of the *I*_D_/*I*_G_ ratio in order to evaluate the degree of perfection of the MWCNTs. Table 1 shows that the G peak was located at the range of 1,573.2 ~ 1,580.1 cm^-1^ and the D peak was located at the range of 1,324.8 ~ 1,328.2 cm^-1^. In this study, two different temperature regions are observed for the oxidation behavior of MWCNTs. Both the G peak and the D peak with minimum wavelengths were revealed in the 300°C-annealed MWCNTs. As Figure 6 shows, the G peak at approximately 1,580 cm^-1^ is the E_2g_^2^ model corresponding to the movement in the opposite direction of two neighboring carbon atoms in a graphitic sheet, and it indicates the presence of crystalline graphitic carbon in MWCNTs. The D peak at approximately 1,325 cm^-1^ is an A_1g_ breathing mode, and this mode is generally attributed to the defects in the curved graphite sheet, sp^3^ carbon, or other impurities.

**Table 1:** **Wavelengths of *I***_**D **_**and *I***_**G **_**peaks and calculation value of *I***_**D**_**/*I***_**G **_**ratio under different treatment temperatures**

	**Temperature**				
	**As-deposited**	**200°C**	**300°C**	**400°C**	**500°C**
*I*_D_ (cm^-1^)	1,328.2	1,325.2	1,324.8	1,326.5	1,327.8
*I*_G_ (cm^-1^)	1,580.1	1,575.5	1,573.2	1,575.7	1,576.4
*I*_D_/*I*_G_ ratio	0.898	0.878	0.484	0.792	0.92

Figure 6 also shows that the D band and the G band had apparent changes in their intensities as the oxidation temperature was increased, and the intensity of the D band peak at approximately 1,620 cm^-1^ increased with increasing oxidation temperature. The *R* = *I*_D_/*I*_G_ ratio, where *I* corresponds to the peak area of the Lorentzian functions, allows us to estimate the relative extent of structural defects. Table 1 shows that the *I*_D_/*I*_G_ ratio of the 200°C-annealed sample was equal to the value of as-received tubes. This result suggests that no oxidation happens on the MWCNTs under this condition. As the oxidation temperature was increased to 300°C, the *I*_D_/*I*_G_ ratio decreased to 0.484. The removal of defective tubes (some amorphous carbon layers, sp^3^ carbon, and other impurities) and improvement of disordered carbon are the reasons [16]. Therefore, as the oxidation temperature was further increased from 300°C to 500°C, the *I*_D_/*I*_G_ ratio induced an increase from 0.484 to 0.92. As the MWCNTs are annealed in oxygen atmosphere, the increase in *I*_D_/*I*_G_ ratio is believed to be caused by the enhancement of surface defects and embedment of oxygen atoms.

**Figure 7:**
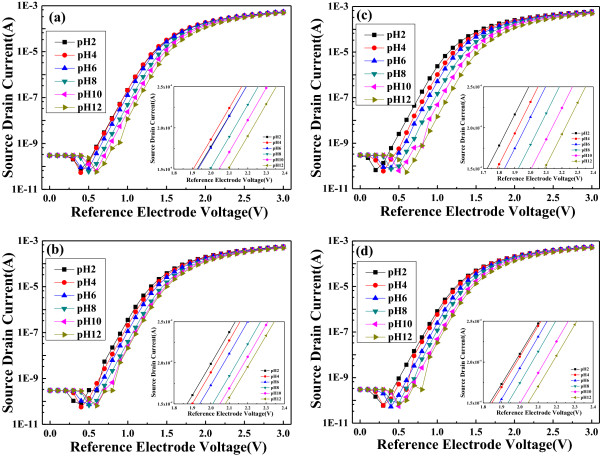
**Current-voltage characteristics of EGFET sensors with different treatment temperatures. (a)** As-deposited, **(b)** 300°C, **(c)** 400°C, and **(d)** 500°C.

As we know, a high-impedance material is suitable for ISFET devices; in contrast with ISFET devices using a high-impedance material as their sensing films, the ion sensing films of EGFET devices are fabricated using a low-impedance material for relatively better conductivity and sensitivity. In this study, the oxidized In (or called In_2_O_3_) films were used as the low-impedance material. Meanwhile, the EGFET devices' structure comprises a MOSFET which retains a metal gate electrode and utilizes a signal wire to connect the separative ion sensing film and the field-effect transistor. Figure 7 shows that the change of conductivity leads to variations of the reference voltages for the MWCNTs/In_2_O_3_ electrode in pH buffer solution using the EGFET devices' structure at pH 2, pH 4, pH 6, pH 8, pH 10, and pH 12. As we know, if In films are annealed in oxygen atmosphere, then they are oxidized into n-type In_2_O_3_ films. For that, the different composition ratios of In_2_O_3_ will cause the change of conductivity and lead to variations of the reference voltages. The sensitivity of the MWCNTs/In_2_O_3_ films is characterized by measuring the electrodes in solutions with various pH values at room temperature when a fixed drain voltage of 0.3 V is selected. We experimentally found that the conductivity (or the variations of the reference voltages) of the MWCNTs/In_2_O_3_ films depended on the pH range of the buffer solution and the oxidation temperature of the MWCNTs/In_2_O_3_ films in the EFGET devices. Due to the OH group that is attached on the wall of the MWCNTs and the surface of In_2_O_3_ films, the pH buffer solutions can increase or decrease the conductivity of the MWCNTs/In_2_O_3_ films. The significant changes in the electronic properties of the MWCNTs/In_2_O_3_ films are caused by the interaction between the hydroxide in the pH solution and the surface of the MWCNTs/In_2_O_3_ films.

**Figure 8:**
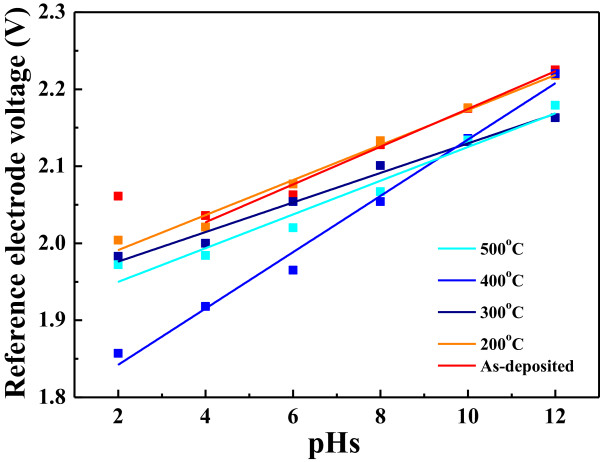
Reference electrode voltage measurement of pH sensors with various thermal temperatures at various pH buffer solutions.

As shown in the insets of Figure 7a,b,c,d, the variations of the reference voltages for the MWCNTs/In_2_O_3_ electrode without thermal treatment did not show linear dependence on the low pH value of the buffer solution, due to the acid corroding the In (In_2_O_3_) films. On the other hand, the reference voltage of the MWCNTs/In_2_O_3_ electrode after thermal treatment was almost linearly dependent on the pH value of the buffer solution, specifically in the measurement of 400°C thermal treatment as shown in Figure 7c. The linear region shown in the insets of Figure 7 can be used to investigate the sensitivity of the MWCNTs/In_2_O_3_ film-formed EGFET devices. The slopes of Δ*I*/Δ*V* shown in the insets of Figure 7a,b,c,d are 3.89 × 10^-4^, 3.73 × 10^-4^, 3.70 × 10^-4^, and 3.62 × 10^-4^ A/V, respectively. These results suggest that the 500°C-annealed MWCNTs/In_2_O_3_ films have the maximum variation as the same variation of current is measured. As we know, the Nernst equation is a mathematical description of an ideal pH electrode behavior in electrochemistry [17]. It can be used to calculate the reduction potential of an electrochemical cell or to find the concentration of one of the components of the cell. The Nernst equation can also accurately predict cell potentials only as the equilibrium quotient is expressed in activities. For that, the linear variations of calculated reference electrode voltage in Figure 8 are assumed as the Nernst equation relating to the total double-layer potential drop in the activity of H^+^ (or OH^-^) in the solution. Recent calculations of the double-layer potential drop for oxides based on a simple model of the oxide/solution interface have shown that the change in double-layer potential drop with pH is generally less than 59.1 mV per pH unit [18,19]. Table 2 depicts the sensitivity of the electrode as a function of the thermal treatment temperature of the MWCNTs/In_2_O_3_ films. The sensitivity first increased with increasing annealing temperature and reached the highest sensitivity of about 36.43 mV/pH for 400°C-annealed MWCNTs-In_2_O_3_ films, which was lower than the theoretical value of 59.1 mV/pH.

**Table 2:** **Sensitivity of MWCNTs/In**_**2**_**O**_**3 **_**sensing layer as a function of thermal treatment temperature**

	**Temperature**				
	**As deposited**	**200°C**	**300°C**	**400°C**	**500°C**
Sensitivity (mv/pH)	16.24	24.06	31.37	36.43	30.94

## Conclusions

In this study, XRD patterns showed that as the annealing temperature was equal to and higher than 400°C, only the In_2_O_3_ phase was clearly observed in the bi-layer MWCNTs-In_2_O_3_ films. The composite MWCNT-In_2_O_3_ electrode was used in the EGFET devices to enhance the performance of pH sensors. From the Raman spectra, as the oxidation temperature was further increased from 300°C to 500°C, the *I*_D_/*I*_G_ ratio (*R*) induced an increase from 0.484 to 0.92. The increase in *R* values was believed to be caused by the enhancement of surface defects and embedment of oxygen atoms. The variation of the reference voltage for the MWCNTs/In_2_O_3_ electrode in the EGFET devices without thermal treatment did not show linear dependence on the low pH value of the buffer solution. The reference voltage of the MWCNTs/In_2_O_3_ electrode after thermal treatment was almost linearly dependent on the pH value of the buffer solution. It was found that the superior sensitivity characteristic of the MWCNT/In_2_O_3_ films in the EGFET devices was 36.43 mV/pH while the thermal treatment temperature was 400°C.

## Competing interests

The authors declare that they have no competing interests.

## Authors' contributions

Dr. S-CH drafted the manuscript. Dr. N-JC participated in the design of the study and performed the statistical analysis. Dr. C-FY conceived of the study, participated in the manuscript's design and coordination, and helped to draft the manuscript. Mr. Y-PL proceeded the immunoassays. All authors read and approved the final manuscript.

## Authors' information

S-CH was born in Taipei, Taiwan. After graduating in electrical engineering from the University of Alabama, Huntsville, he returned to Taiwan and worked at MATRA (France) branch in Taiwan as an electrical engineer responsible for constructing the first subway in Taiwan in 1992. He is now an associate professor at Shih Chien University, Kaohsiung, Taiwan. Much of Hung's research interests has been in the field of one-dimensional nanostructures including the design, fabrication, and characterization of optoelectronic materials for device applications. He is also in the field of carbon nanotubes for pH sensor application and announced in 2012 to 2014.

N-JC graduated from the Department of Physics, National Cheng Kung University in 1988. He obtained his master's degree and PhD degree from the Department of Optics and Photonics, National Central University in 1992 and 1999. After obtaining his master's degree, he joined the Digital Signal Processing Division of Chunghua Telecom Laboratories in 1994 as an assistant researcher. He is currently an assistant professor in the Institute of Photonics and Communications at National Kaohsiung University of Applied Sciences, Kaohsiung, Taiwan. His research interests involve physics education, optical information processing, optical metrology, image processing, and 3-D optical profilometry.

C-FY gained his bachelor's, master's, and PhD degrees in 1976, 1988, and 1993, respectively, from the Department of Electrical Engineering of Cheng Kung University. After obtaining his PhD degree, Yang entered the Department of Electronic Engineering, Chinese Air Force Academy and since February 2000 as a professor at the Chinese Air Force Academy, Taiwan. In February 2004, he became a professor of Chemical and Materials Engineering at National University of Kaohsiung (NUK). His current research interests are focused on fine ceramics, microwave ceramics, dielectric thin films, optical materials, transparent conducting oxides, solar cell materials, applications of carbon nanotubes, microwave antennas, and microstrip filters.

Y-PL was born in Taiwan. He got his master's degree in the Graduate Institute of Electro-Optical Engineering and Department of Electronic Engineering, National Taiwan University of Science and Technology, Taipei, Taiwan. While furthering his graduate program, Lo focuses his research on composite structures with carbon nanotube nanomaterials for pH sensor application.
